# Real-time individual benefit from social interactions before and during the lockdown: the crucial role of personality, neurobiology and genes

**DOI:** 10.1038/s41398-022-01799-z

**Published:** 2022-01-21

**Authors:** Maximilian Monninger, Pascal-M. Aggensteiner, Tania M. Pollok, Iris Reinhard, Alisha S. M. Hall, Lea Zillich, Fabian Streit, Stephanie-H. Witt, Markus Reichert, Ulrich Ebner-Priemer, Andreas Meyer-Lindenberg, Heike Tost, Daniel Brandeis, Tobias Banaschewski, Nathalie E. Holz

**Affiliations:** 1 Department of Child and Adolescent Psychiatry and Psychotherapy, Central Institute of Mental Health, Medical Faculty Mannheim/Heidelberg University, J5, Mannheim, 68159 Germany; 2 Department of Biostatistics, Central Institute of Mental Health, Medical Faculty Mannheim/Heidelberg University, J5, Mannheim, 68159 Germany; 3 Department of Genetic Epidemiology in Psychiatry, Central Institute of Mental Health, Medical Faculty Mannheim/Heidelberg University, J5, Mannheim, 68159 Germany; 4 Department of Psychiatry and Psychotherapy, Central Institute of Mental Health, Medical Faculty Mannheim/Heidelberg University, J5, Mannheim, 68159 Germany; 5 mental mHealth lab, Institute of Sport and Sports Science, Karlsruhe Institute of Technology, Engler-Bunte Ring 15, 76131 Karlsruhe, Germany; 6 Department of Child and Adolescent Psychiatry and Psychotherapy, Psychiatric Hospital, University of Zurich, Neumünsterallee 9, Zurich, 8032 Switzerland; 7 Center for Integrative Human Physiology, University of Zurich, Winterthurerstr. 190, Zurich, 8057 Switzerland; 8 Neuroscience Center Zurich, University of Zurich and ETH Zurich, Winterthurerstr. 190, Zurich, 8057 Switzerland; 9 Donders Institute, Radboud University, Nijmegen, the Netherlands; 10 Radboud University Medical Centre, Nijmegen, the Netherlands

**Keywords:** Human behaviour, Schizophrenia, Neuroscience

## Abstract

Social integration is a major resilience factor for staying healthy. However, the COVID-19-pandemic led to unprecedented restrictions in social life. The consequences of these social lockdowns on momentary well-being are yet not fully understood. We investigated the affective benefit from social interactions in a longitudinal birth cohort. We used two real-time, real-life ecological momentary assessments once before and once during the initial lockdown of the pandemic (*N* = 70 participants; *n*~6800 observations) capturing the protective role of social interactions on well-being. Moreover, we used a multimethod approach to analyze ecological assessment data with individual risk and resilience factors, which are promising moderators in the relationship of social behavior, stress reactivity, and affective states (i.e., amygdala volume, neuroticism, polygenic risk for schizophrenia). Social contacts were linked to higher positive affect both during normal times and during the COVID-19-pandemic (beta coefficient = 0.1035), highlighting the beneficial role of social embedding. Interestingly, this relationship was differentially moderated by individual risk and resilience factors. In detail, participants with a larger left amygdala volume (beta coefficient = −0.0793) and higher neuroticism (beta coefficient = −0.0958) exhibited an affective benefit from more social interactions prior to the pandemic. This pattern changed during the pandemic with participants with smaller amygdala volumes and lower neurotic traits showing an affective gain during the pandemic. Moreover, participants with low genetic risk for schizophrenia showed an affective benefit (beta coefficient = −0.0528) from social interactions irrespective of the time point. Our results highlight the protective role of social integration on momentary well-being. Thereby, we offer new insights into how this relationship is differently affected by a person’s neurobiology, personality, and genes under adverse circumstances.

## Introduction

At the beginning of 2020, the coronavirus (COVID-19) outbreak was declared a global pandemic by the World Health Organization (WHO). In addition to posing a dramatic public health burden, the pandemic also brought drastic social contact restrictions (“*lockdown”)*, which began on the 23rd of March 2020 in Germany. During this lockdown period, individuals were only allowed to meet with people from one other household. First studies from China investigating the mental health outcomes of the pandemic already demonstrated initial evidence for elevated levels of stress, anxiety, and depressive symptoms [1, 2]. By now, further population-based and longitudinal studies comparing well-being before and at different stages during the pandemic found constant elevations of mental health problems, increased levels of internalizing and externalizing symptoms, heightened distress, increased feelings of loneliness, enlarged sleep difficulties, and reduced quality of life measures in different age groups [3–6]. Moreover, several studies point to symptom worsening effects of the COVID-19 pandemic in various clinical samples partially due to limited access to health care providers or a lack of social integration [7, 8].

Several studies conducted before the COVID-19 pandemic indicated that social integration (e.g., the amount of social interactions, the social network size, self-perceived social support) plays an important role in promoting resilience and momentary well-being [9–13]. However, little is known about how social contact restrictions due to the pandemic alters this relationship. To our knowledge, there is only one published study comparing social network characteristics prior to and during the lockdown phase from a longitudinal perspective: In a Swiss student sample, participants reported significantly fewer interactions and study partners on average during than before the lockdown, although friendships and perceived social support did not change [14], indicating that structural characteristics (i.e., the objective quantity of social interactions) are more impaired due to the lockdown than evaluative and subjective characteristics of social relationships (i.e., the self-perceived quality of a social interaction). Critically, this study did not address the direct effect of social interactions on momentary affective states. Therefore, we aimed at investigating the specific relationship between social contacts and momentary well-being both before and during the lockdown phase in Germany using a real-time, real-life approach in the framework of a longitudinal study.

Moreover, so far there is a lack in understanding how the relationship between social integration and mental health is determined by an individuals’ risk and resilience profile, with initial findings suggesting an involvement of neurobiological structure, personality traits, and genetic make-up.

Besides others, the amygdala is a core structure of the social brain [15] and has been shown to be affected by socioenvironmental influences in general [16] and by the COVID-19 pandemic in particular [17]. It has been proven as a key convergence site of social adversity [18] and is strongly involved in stress adaptation [18–21]. In addition, several studies reported its prominent role in the processing of emotions and thereby its involvement in the etiology and persistence of mood disorders [22, 23]. Moreover, previous studies found a positive relationship between larger amygdala volumes and increased social network sizes [24, 25], heightened perceived social support [26], and social connectedness [27], indicating a key role of the amygdala in how a person is socially integrated. Taken together, it is reasonable to assume a potentially moderating function of the amygdala in the impact of social environmental influences and social behavior on current well-being, which, however, has not been tested before.

In terms of personality, high levels of neuroticism are correlated with diminished social integration, including lower frequency of involvement in real-life social interactions, a smaller social network, and an overall weaker social bonding [28]. In addition, neuroticism is linked to heightened risk of mood disorders, elevated stress reactivity, overestimation of potential health threats [29–31], and self-reported changes in social behavior due to the COVID-19-pandemic [32]. Furthermore, higher neurotic traits significantly predicted decreased emotional, psychological, and social well-being during the COVID-19 pandemic and poorer mental health outcomes [32, 33]. In summary, previous findings indicate that individuals scoring high on neurotic traits are less equipped with social coping skills, tend to more social withdrawal, and heightened levels of negative affective states. However, it remains unclear, how personality traits, such as neuroticism, shape an individuals’ momentary social behavior, which may have consequences on their affective states. To address this gap in the literature, we used neuroticism as an additional candidate moderator in the relationship of social interactions on momentary well-being.

Finally, while there is a long tradition on the research of the role of heritability and gene-environment interactions in psychiatric disorders in general, findings on genetic influences on momentary behavior or current affective states are largely missing. For instance, several studies so far reported cumulative evidence for the prominent role of genetics in the etiology of schizophrenia. As such, the genetic risk for schizophrenia has been highlighted as conferring a maladaptation to social contexts [34, 35], with patients suffering from schizophrenia often characterized by weak social integration, a reduced social network size, and fewer friends [36, 37]. Moreover, increased polygenic risk scores for schizophrenia are associated with heightened risk for further psychiatric disorders, including anxiety, mood, and personality disorders [38], which are also characterized by social withdrawal and lower affective states. In addition, schizophrenia has previously been linked to higher affective reactivity to daily stressors [39], suggesting a heightened sensitivity to the COVID-19 pandemic in individuals with a higher polygenic risk for schizophrenia (SCZ-PRS). Given previous findings, it is reasonable to assume that an individuals’ genetic make-up might be critical in modulating the direct sensitivity to the social environment and its immediate impact on well-being, which has not been tested before. Therefore, we further investigated the above-mentioned risk and resilience markers that are based on previous findings specifically qualified to modulate how social interactions influence momentary well-being.

Within the framework of an ongoing longitudinal birth-cohort at risk (“Mannheim Study of Children at Risk”), we assessed amygdala volume, neuroticism, and SCZ-PRS prior to the pandemic and social interactions along with well-being using EMA before and during the lockdown. Based on previous findings [13] and on the protective role of social interactions when adversity is encountered [16], we expected to find a strong positive association between social interactions and well-being both prior to and during the pandemic. Moreover, our approach allowed us to further investigate the moderating effects of amygdala volume, neuroticism, and SCZ-PRS on the impact of social interactions on momentary well-being. Given the role of the amygdala, neuroticism, and SCZ-PRS in socioenvironmental risk and resilience [24, 31, 34, 39–41], we expected individuals with larger amygdalae volumes, lower neurotic traits, and lower SCZ-PRS to show an affective benefit from social interactions and tested whether this is different under social contact restrictions.

## Materials and methods

### Sample

The present investigation was conducted in the framework of the Mannheim Study of Children at Risk (‘MARS’), an ongoing prospective study of the long-term outcomes of early psychosocial and biological risk factors following children since birth. The initial sample consisted of 384 children born between 1986 and 1988 in the Rhine-Neckar region of Germany and were included according to a two-factorial design intended to enrich and control the risk status of the sample (see [42] for full details).

Starting at the age of 3 months, information on mental health, personality traits, and genetic variability was collected prospectively up to the most recent assessment wave at the age of 32–33 years, which was disrupted by the COVID-19 pandemic. This assessment consisted of a questionnaire package on physical and mental health, a diagnostic interview, MRI measurements, and an EMA. Starting shortly after the social contact restrictions were put in place in Germany in April 2020, participants who had completed the EMA week (*n* = 165) were invited to take part in an online survey and to repeat the EMA procedures during the COVID-19 pandemic. A total of 133 participants completed the online survey and 70 participated (44 female; mean age = 33.36 years; distribution in the current sample: 22 (31.4%) participants without psychosocial risk, 26 (37.1%) with low psychosocial risk, and 22 (31.4%) with high psychosocial risk at birth) in both EMA measurements. The study was approved by the Ethics Committee of the University Heidelberg, Germany, written informed consent from all participants was obtained, and participants were financially compensated.

### EMA procedures

Participants were asked to install a commercial e-diary app (MovisensXS, version 1.4.3) on their own Android smartphone. The e-diary started on a fixed date, with the participants receiving prompts via an acoustic, visual, and vibration signal. Prompts were scheduled from 8 am until 10 pm with a fixed interval of 120 min to facilitate retrospection, resulting in a maximum of eight prompts per day and 56 prompts per week. Upon receiving a prompt, participants completed the questionnaire, which took ~90 s. Participants had the opportunity to postpone a prompt for a maximum of 25 min. If participants did not respond within the 25 min to a prompt, a missing was noted. The same procedure was repeated during the lockdown phase of the COVID-19 pandemic in Germany beginning on 23 April 2020 (Supplementary Fig. 1).

### Affective state

Positive and negative affect was measured using a 15-item short version of the German adaptation of the Positive and Negative Affect Schedule (PANAS) [43] with additional items capturing stress reactivity [44–47]. Participants were asked to rate their current positive or negative feelings on a 7-point Likert scale (1 = fully disagree, 7 = fully agree). Mean scores for positive and negative affect were calculated for each prompt and used in all analyses as dependent variables. Between- and within-person reliability coefficients for positive (*R*_kf_ = 0.99; *R*_cn_ = 0.62) and negative affect (*R*_kf_ = 0.99; *R*_cn_ = 0.75) were calculated using mixed models [48] and ranged from moderate to high.

### Momentary social contacts

Participants were asked to indicate the number of real-life social contacts and the quality of the most important interaction within the last 2 h before the prompt (i.e., the interval between two prompts). Real-life social contacts are defined as interactions in which participants were talking to or interacting with another person face-to-face. For the quality rating, participants were asked to indicate on a visual analogue scale ranging from 0 to 100 how positive the most important interaction was experienced (0 = very negative, 100 = very positive). If participants reported no interactions within the current time-frame, no follow-up question was presented.

### Stress burden during COVID-19

We used two items rated on a 10-point Likert scale (0: very low; 10: very high) to assess the impact of COVID-19 on physical and mental health (*‘The physical burden of COVID-19 for me is…’, ‘The mental burden of COVID-19 for me is…’*).

### Moderator variables

We tested for a possible moderating impact of the bilateral amygdala volume (*n* = 70), neuroticism (*n* = 69), and SCZ-PRS (*n* = 68) on the relationship of social interactions with positive and negative affect.

### Amygdala volume

At the age of 32–33 years and prior to the COVID-19 pandemic, high-resolution anatomical images with 208 slices covering the whole brain were acquired using a 3T-scanner (PrismaFit; Siemens) with a standard 32-channel head coil. Volumetric segmentation was performed with the FreeSurfer image analysis suite (Version 6.0.0) as described previously [49] to indicate left and right amygdala volume (mm^3^).

### Personality traits

Neuroticism was assessed at the age of 25 years using the German version of the NEO Five-Factor Inventory (NEO-FFI) [50], a widely used instrument to determine the Big Five personality traits. It contains a total of 60 items, with 12 items for each personality trait, rated on a 5-point Likert scale (1 = strongly disagree, 5 = strongly agree). Sum scores for neuroticism were calculated, with higher values representing a higher trait expression.

### Polygenic risk scores

DNA was extracted from whole blood or saliva of 306 participants of the initial sample. Genome-wide genotyping was performed using Global Screening Array 24 version 2 (Illumina, Inc., San Diego, CA, USA) at the Life & Brain facilities, Bonn, Germany. Quality control and filtering was performed using PLINK v1.90b6.7 [51], removing participants with >0.02 missingness, heterozygosity rate > |0.20|, and sex-mismatch. SNPs with a minor allele frequency of <0.01, deviating from Hardy-Weinberg equilibrium (HWE) with a *p*-value of <10^−6^ and missing data > 0.02 were removed. Relatedness and population structure were filtered based on a SNP set filtered for high quality (HWE *P* > 0.02, MAF > 0.20, missingness = 0), and LD pruning (*r*² = 0.1). If subjects were cryptically related (pi hat > 0.10), one subject was excluded at random. Control for population stratification was performed by generating principal components and outliers, defined as deviating more than 4 SD on one of the first 20 principal components were excluded. Quality control and filtering resulted in a data set of 301 individuals and 482,981 SNPs. Of those, 68 subjects with available EMA data were included in the present analyses.

SCZ-PRS were calculated based on the genome-wide association data of 77,096 individuals (33,640 cases, 43,456 controls) from the Psychiatric Genomics Consortium (PGC) for SCZ [52]. SCZ-PRS were calculated for 68 participants of the present study with PRSice version 2.2.6 [53] after clumping SNPS (linkage disequilibrium *r*² < 0.1 250 kb sliding window) for multiple *p*-value thresholds. For the analysis in the present study, we selected the PT = 0.05, as this was the threshold with the best prediction in the discovery samples.

### Covariates

Gender, time of day, estimated intracranial volume (ICV), and the first ten principal components (to control for population stratification) were included as covariates when applicable. To ensure that the level of stress-dependent changes in positive affect were not due to biological programming by early postnatal stressful environments or encountered stress during lifetime, we additionally controlled for psychosocial risks at birth and stressful life events over the lifespan.

### Psychosocial risk

Psychosocial risk was assessed using a standardized interview according to an enriched family adversity index [54] at the participants´ age of 3 months, covering 11 items of the family environment, the parents, and their partnership (e.g., parental psychiatric disorders, overcrowding, or ongoing parental conflicts). A sum score of psychosocial risks were calculated by adding up the presence of all items.

### Stressful life events

Life events were recorded using a modified version of the Munich Events List (MEL; [55]) starting at the first assessment wave at the age of 3 months until the last assessment wave prior the COVID-19 pandemic. The MEL covered several areas of acute and chronic, positive and negative stressors, which were adjusted for different developmental stages. (e.g., school entrance at the age of 8 years; university entrance at the age of 19 years, but also chronic illness of a relative, ongoing parental disharmony or loss of a family member). Sum scores for each assessment wave were added to calculate an overall life events score.

### Data analysis

A-priori performed power calculations using the freely available R package EMAtools [56] revealed 80% power to detect medium-sized effects (*d* = 0.5) with a compliance rate above 85% based on a sample size of 70 participants with 14 observation days and a maximum of 8 responses per day.

Multilevel analyses were conducted to analyze the association between the quantity of social interactions (i.e., the number of social interactions) and current affective states as well as the statistical interaction with time point (pre- and during COVID-19 pandemic) and the potential moderators amygdala volume, neuroticism, and SCZ-PRS. Momentary affective states acted as the dependent variable and the number of social contacts as predictor variable (level-1), which were person-mean centered and nested within participants (level-2). Amygdala volume and neuroticism as level-2 variables were grand-mean centered. In addition, for visualization purposes, SCZ-PRS were z-standardized. To investigate the impact of COVID-19, a dichotomous time point variable (0 = pre-COVID-19, 1 = during-COVID-19) was included in all multilevel models. Furthermore, covariates of no interest, consisting of gender, time of day, psychosocial risk factors, ICV, and the first ten principal components of population stratification, when applicable, were entered in all models. Psychosocial risk factors, stressful life events, and ICV were grand-mean centered, whereas time of day was calculated in hours by subtracting the daily start time (i.e., 8 am) from all values. In addition, the aggregated person-means for real-life social contacts were entered in all models separately to control for their potential effects.

We fitted five mixed models including random intercepts as well as random slopes for all level-1 predictors. In Model-I, we included real-life social contacts and time point as main effects of interest, and the corresponding two-way interaction effects. Models-II to V were based on Model-I, but additionally included one of the potential moderators at a time, i.e., left or right amygdala volume, neuroticism, or SCZ-PRS as a main effect, together with the corresponding two-way and three-way interaction effects. In a sub-analysis, we specified the effect of the quality of the most important interaction as an additional predictor (level-1, person-mean centered) for momentary affect with the corresponding two-way and three-way interaction effects. Given that the quality of social interaction was only assessed if social interactions have been indicated, resulting in fewer observations (*N* = 5820), this model had a lower power and is thus considered exploratory (see Supplement for further analyses with the quality of social interactions as additional predictor). All multilevel models were designed with the freely available R packages lme4 [57], and lmerTest [58] to compute *p*-values. To further analyze the interaction effects, simple slope analyses and Johnson-Neyman plots were computed in order to estimate the range of values of the moderator variable in which the slope of the predictor is significant vs. nonsignificant. For all analyses, the two-sided alpha level was Bonferroni-corrected set at 0.01 (*p* = 0.05 divided by 5 models). As a measure to compare the magnitude of effects, standardized beta coefficients were computed for all multilevel models following established procedures [59]. The custom code used for the analyses of this study is available from the corresponding author upon reasonable request.

## Results

### Descriptive data

In total, 6837 prompts were answered by 70 participants (63% female; mean age = 33.36 years) across both time points. On average, participants responded to 97.9 prompts (SD = 12.18; range = 57–112; baseline: mean = 49.86 prompts, COVID-19: mean = 48.04 prompts, Supplementary Table 1), resulting in a high compliance rate of 87.41% across both time points. The earliest start date of the assessment was 61.43 weeks prior to the social contact restrictions in Germany (23 March 2020) for the baseline data (latest inclusion date for baseline data 2 March 2020) and 4.57 weeks (32 days) after the restrictions were put in place for the COVID-19 assessment. As expected, participants reported significantly more weekly real-life contacts (mean = 3.89; SD = 2.81) in the baseline assessment compared to the COVID-19 assessment (mean = 2.50; SD = 1.37; *t*_138_ = 3.734, *P* < 0.001), while the quality of interactions (mean = 76.15; SD = 18.27, range = 0–100) did not change (*P* = 0.81). Given that only the quantity of social interactions changed between time points, we focused on this variable in the moderation analyses.

Overall, while positive affect decreased during COVID-19 (*P* < 0.001), momentary negative affect was higher (*P* = 0.005). Additional linear regression analyses revealed that COVID-19-related stress predicted lower positive affect, which did not reach significance after correction for multiple testing (*P* = 0.038). Moreover, this did not pertain to social contact reductions (*P* = 0.664) or negative affect (*P* > 0.8).

### Social contacts and affective state (Model-I)

**Fig. 1:**
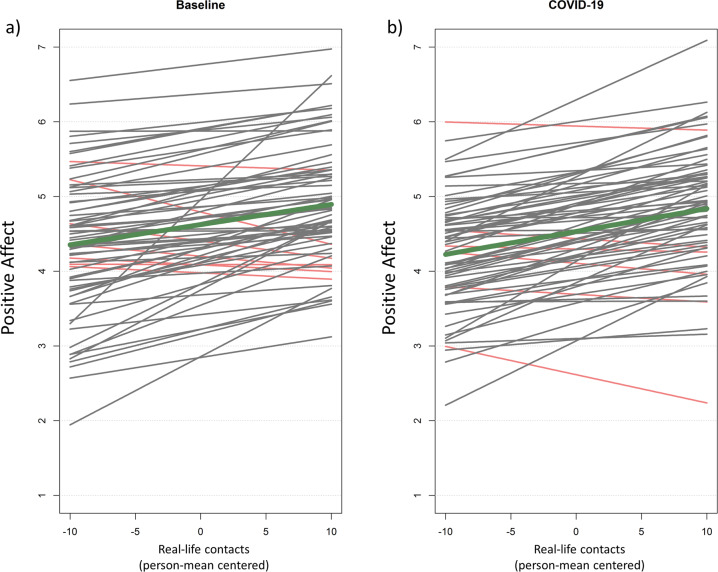
Individual social affective benefit. Individual associations of real-life contacts and positive affect during baseline (**a**) and during COVID-19 (**b**). Real-life contacts represent person-mean centered social contacts within the last 2 h. Differences from zero indicate an increased/decreased amount of social contacts compared to the person-mean. Gray and red lines reflect positive and negative slope values, respectively. Thick line in dark green reflects the association for the whole group. Notably, decreased overall positive affect during the COVID-19 pandemic was not fully explained by social contact reductions.

The number of real-life contacts and the quality of the most important interaction were significantly associated with positive affect across both time points (beta coefficient = 0.0693; *P* = 0.001; Supplementary Table 2a, b, Fig. 1), indicating a mood-uplifting effect by social interactions. In addition, a more pleasant social interaction was associated to lower negative affect (beta coefficient = −0.01545; *P* < 0.001, Supplementary Table 3b), however, no such relationship was found regarding the quantity of real-life contacts (beta coefficient = 0.0142; *P* = 0.540; Supplementary Table 3a). Therefore, no further analyses with negative affect as outcome were performed.

### Amygdala volume, social contacts, and affective state (Models-II and III)

Left but not right amygdala volume moderated the relationship between the number of social contacts, time point and positive affect (beta coefficient = −0.0793; *P* = 0.008, Table 1; beta coefficient = −0.0139; *P* = 0.675, Supplementary Table 4a, respectively).

**Table 1:** Mixed model results for social contacts, left amygdala volume, and positive affective states across both time points.

	Positive affect				
Predictors	Estimates	std. Beta	CI	Standardized CI	*p*
(Intercept)	4.7326	0.0400	4.1635–5.3017	−0.1694–0.2495	<0.001
ICV	0.0017	0.2208	0.0002–0.0033	0.0212–0.4204	0.030
Stressful life events	−0.0015	−0.0318	−0.0100–0.0071	−0.2187–0.1551	0.739
Psychosocial risk at birth	−0.0371	−0.0664	−0.1373–0.0632	−0.2461–0.1132	0.469
Gender	0.0154	0.0154	−0.3470–0.3778	−0.3463–0.3771	0.934
Time of day	−0.0003	−0.0014	−0.0081–0.0075	−0.0369–0.0340	0.937
Time point	−0.0726	−0.0713	−0.1078–−0.0374	−0.1064–−0.0362	<0.001
Left amygdala volume	−0.0009	−0.1623	−0.0020–0.0002	−0.3611–0.0366	0.110
Momentary real-life contacts	0.0221	0.1056	0.0095–0.0347	0.0449–0.1664	0.001
Aggregated real-life contacts	0.1248	0.2118	0.0330–0.2167	0.0559–0.3676	0.008
Left Amygdala * Time point	−0.0003	−0.0518	−0.0005–−0.0001	−0.0866–−0.0171	0.003
Momentary real-life contacts * Time point	0.0005	0.0044	−0.0114–0.0125	−0.0530–0.0618	0.930
Momentary real-life contacts * Left amygdala volume	0.0001	0.0409	−0.0000–0.0001	−0.0210–0.1029	0.195
Momentary real-life contacts * Left amygdala volume * Time point	−0.0001	−0.0793	−0.0002–−0.0000	−0.1379–−0.0206	0.008
**Random effects**					
σ^2^	0.4911				
τ_00 Participants_	0.4988				
τ_11 Time of day_	0.0008				
τ_11 Real-life contacts_	0.0017				
ICC	0.50				
N _VPNr_	70				
Observations	6837				
Marginal *R*^2^/Conditional *R*^2^	0.105/0.545				

**Fig. 2:**
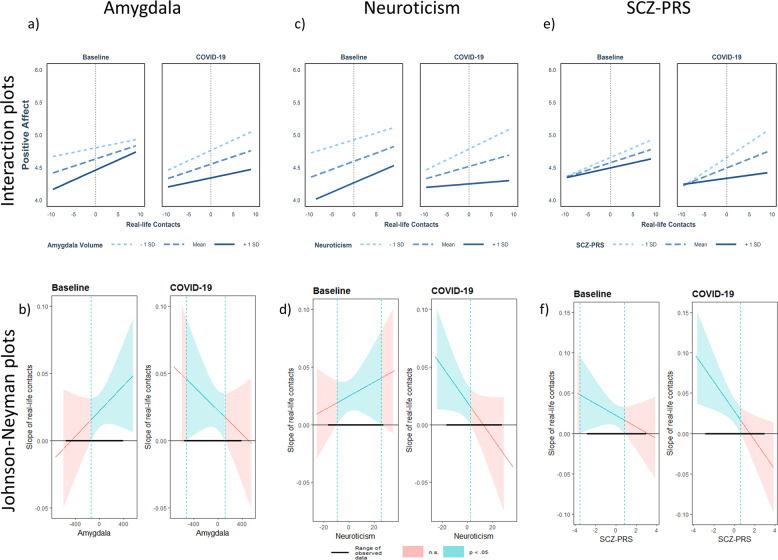
Interaction- and Johnson-Neyman plots for all significant three-way interactions with the number of real-life contacts. Top: Plots depicting the interaction between real-life contacts, time point and amygdala (**a**), neuroticism (**c**), and polygenic risk for schizophrenia (SCZ-PRS, **e**) on positive affect. Real-life contacts represent person-mean centered social contacts within the last 2 h. Differences from zero indicate an increased/decreased amount of social contacts compared to the person-mean. Bottom: Johnson-Neyman plots for the significant three-way interactions with amygdala (**b**), neuroticism (**d**), and SCZ-PRS (**f**). Johnson-Neyman plots indicate the range of observed values of a moderator, for which the association (i.e., ‘slope of real-life contacts’) between real-life contacts and positive affect is significant (*p* < 0.05).

Subsequent simple slope analyses and Johnson-Neyman plots (Fig. 2a, b) for the left amygdala showed a significant positive association (i.e., a positive slope) between real-life contacts and positive affect depending on the amygdala size, indicating an affective gain from social interactions only in those with larger amygdala volumes. In contrast, the opposite relationship was present during the lockdown, with an affective gain only in those with average to smaller amygdala volumes.

### Neuroticism, social contacts, and affective state (Model-IV)

The results revealed significant three-way interactions between neuroticism, time point, and the number of real-life contacts (beta coefficient = −0.0958; *P* = 0.003, Table 2). Subsequent analyses for real-life contacts indicated an affective benefit from social interactions irrespective of trait neuroticism before the pandemic. However, during the COVID-19 assessment, the beneficial effect of real-life contacts on positive affect was only significant in those with low neuroticism scores, whereas those with high neurotic traits showed no affective benefit (Fig. 2c, d).

**Table 2:** Mixed model results for social contacts, neuroticism, and positive affective states across both time points.

	Positive affect				
Predictors	Estimates	std. Beta	CI	Standardized CI	*p*
(Intercept)	4.2275	0.0191	3.6847–4.7703	−0.1650–0.2033	<0.001
Stressful life events	0.0068	0.1475	−0.0020–0.0155	−0.0438–0.3388	0.131
Psychosocial risk at birth	−0.0686	−0.1261	−0.1592–0.0221	−0.2929–0.0406	0.138
Gender	0.0503	0.0512	−0.2290–0.3295	−0.2334–0.3359	0.724
Time of day	−0.0001	−0.0001	−0.0080–0.0079	−0.0369–0.0366	0.995
Time point	−0.0701	−0.0707	−0.1050–−0.0352	−0.1063–−0.0351	<0.001
Neuroticism	−0.0408	−0.3909	−0.0584–−0.0232	−0.5599–−0.2220	<0.001
Momentary real-life contacts	0.0247	0.1170	0.0112–0.0381	0.0535–0.1804	<0.001
Aggregated real-life contacts	0.0851	0.1426	0.0004–0.1698	0.0007–0.2845	0.049
Neuroticism * Time point	0.0054	0.0514	0.0017–0.0091	0.0159–0.0869	0.005
Momentary real-life contacts * Time point	−0.0053	−0.0264	−0.0175–0.0069	−0.0840–0.0313	0.396
Momentary real-life contacts * Neuroticism	0.0006	0.0271	−0.0009–0.0021	−0.0382–0.0924	0.417
Momentary real-life contacts * Neuroticism * Time point	−0.0022	−0.0958	−0.0036–−0.0008	−0.1579–−0.0336	0.003
**Random effects**					
σ^2^	0.4748				
τ_00 Participants_	0.3913				
τ_11 Time of day_	0.0008				
τ_11 Real-life contacts_	0.0020				
ICC	0.47				
N _VPNr_	69				
Observations	6736				
Marginal *R*^2^/Conditional *R*^2^	0.153/0.544				

### Polygenic risk scores, social contacts, and affective state (Model-V)

Finally, there was a three-way interaction for SCZ-PRS, quantity of social contacts, and time point on positive affect (beta coefficient = −0.0528; *P* = 0.036, Table 3). Subsequent analyses revealed that those with low to moderate SCZ-PRS showed an affective benefit from social interactions before COVID-19 (Fig. 2e, f). This relationship between real-life contacts and positive affect even increased during COVID-19 in participants with low to moderate SCZ-PRS.

**Table 3:** Mixed model results for social contacts, SCZ-PRS, and positive affective states across both time points.

	Positive affect				
Predictors	Estimates	std. Beta	CI	Standardized CI	*p*
(Intercept)	4.4443	−0.0219	3.8505–5.0381	−0.2328–0.1890	<0.001
Stressful life events	0.0015	0.0331	−0.0074–0.0104	−0.1612–0.2273	0.739
Psychosocial risk at birth	−0.0964	−0.1731	−0.2094–0.0166	−0.3760–0.0299	0.095
Gender	0.1646	0.1637	−0.1710–0.5003	−0.1700–0.4974	0.336
Time of day	0.0005	0.0023	−0.0074–0.0084	−0.0332–0.0378	0.897
Time point	−0.0791	−0.0786	−0.1147–−0.0434	−0.1140–−0.0432	<0.001
SCZ-PRS	−0.0793	−0.0787	−0.2521–0.0935	−0.2503–0.0928	0.368
Momentary real-life contacts	0.0225	0.1092	0.0094–0.0356	0.0458–0.1727	0.001
Aggregated real−life contacts	0.0951	0.1611	−0.0097–0.1999	−0.0164–0.3387	0.075
SCZ-PRS * Time point	−0.0805	−0.0799	−0.1157–−0.0452	−0.1149–−0.0449	<0.001
Momentary real-life contacts * Time point	0.0051	0.0247	−0.0072–0.0173	−0.0348–0.0841	0.416
Momentary real-life contacts * SCZ-PRS	−0.0072	−0.0348	−0.0199–0.0056	−0.0966–0.0270	0.270
Momentary real-life contacts * SCZ-PRS * Time point	−0.0109	−0.0528	−0.0211–−0.0007	−0.1021–−0.0035	0.036
**Random effects**					
σ^2^	0.4899				
τ_00 Participants_	0.5162				
τ_11 Time of day_	0.0008				
τ_11 Real-life contacts_	0.0018				
ICC	0.51				
N _VPNr_	68				
Observations	6646				
Marginal *R*^2^/Conditional *R*^2^	0.140/0.576				

## Discussion

To the best of our knowledge, this is the first longitudinal study to investigate the social affective benefit in a real-time, real-life setting prior to and during the COVID-19 lockdown. Our results indicate that both the quantity and the quality of social interactions were significantly associated with positive affect, irrespective of the assessed time point (Model I). In addition, we found for the first time that amygdala volume (Model II-III), neuroticism (Model IV), and SCZ-PRS (Model V) moderated the relationship between the number of social contacts and positive affect differently during normal times as compared to the lockdown.

In line with previous findings [13], the number and quality of real-life social interactions exerted mood-uplifting effects, both before and during the lockdown, indicating an overall protective role of frequent social contacts on well-being. Thereby, we critically expand those findings by showing that this relationship is stable even under social contact restrictions.

Moreover, our findings suggest that the beneficial impact of frequent social interactions on well-being might be modified by an individuals’ risk and resilience profile. As such, left amygdala volume (Model-II) moderated the social affective gain differently before and during Covid-19. This finding is in accordance with the current consensus embedding the amygdala in stress adaptation [60] and a left-lateralized sustained involvement in emotional contexts [61–63]. Here, we extend these previous findings by suggesting that one possible mechanism underlying stress adaptation pertains to left amygdala-dependent modulation of the beneficial effect of social interactions. In line with previous reports linking larger amygdala volumes to increased social network sizes and to higher levels of social support [24, 26], only participants with larger amygdala volumes showed a social affective benefit before the lockdown, indicating that these individuals might more actively seek for social interactions to improve positive emotionality. However, this pattern changed under social contact restrictions, with only participants with average to low left amygdala volumes benefiting from more real-life contacts during the lockdown phase of the COVID-19 pandemic. In this regard, it seems that individuals with a larger left amygdala volume may no longer offset their lower positive affect by increasing social contacts under stress when compared to normal times. This may provide a mechanistic understanding of how a larger amygdala volume might be considered a risk marker, which may only manifests under adversity.

Similarly to those with a larger amygdala volume, participants with higher neurotic traits might benefit from more social contacts (Model IV) before Covid-19, thus may catching up on their affective backlog [64]. Interestingly, this pattern was reversed during the lockdown, indicating an expected mood-lifting effect of social interactions only in persons with low to moderate levels of neuroticism. This is consistent with the idea that individuals with high neurotic traits experience uneasy times as more negative and aversive and are less equipped with adequate coping strategies, such as seeking for social support [29]. We speculate that participants with higher neurotic traits show more interactions focused on COVID-19 related information. That is in line with previous studies during the COVID-19 pandemic reporting higher neurotic traits to be associated with decreased overall well-being during the COVID-19 pandemic [32]. Therefore, our results suggest that high levels of neuroticism might be particularly maladaptive on social affective gain during times of crisis, while their impact on the social affective benefit might be less manifest during normal times.

Finally, we found that the genetic risk for schizophrenia moderated the relationship between social contacts and positive affect (Model-V). As expected and in line with previous findings [39], we found that only those with lower genetic risk for schizophrenia showed an affective gain from social interactions. Notably, while this has been previously shown during normal times [39], our results show that this might be aggravated under social contact restrictions. Therefore, enhancing coping strategies in those at risk might prove beneficial to prevent the onset of psychotic, particularly negative, symptoms in individuals at risk. However, this analysis has to be considered exploratory, given that it fell short of significance after multiple comparison correction, is currently based on summary statistics published in 2014 [52], and fails to reach significance with the updated summary statistics (see supplement) [65].

Some limitations of the present study need to be addressed. Since the study started during social contact restrictions, only a quarter of our MARS participants were able to take part in this follow-up measurement. However, the extremely high compliance rate of 87% guaranteed enough power and the sample was not systematically biased regarding demographics and psychosocial risk factors when compared to the dropout sample. Moreover, while our sample is representative regarding socio-demographics and socio-economic factors for the German general population within this age range [66] (see supplement for details), children and adolescents as well as older adults could be affected by the consequences of social isolation in different ways. Therefore, our results show limited generalizability to broader age-ranges. In addition, we used the total number of social interactions without assessing the exact number of specific interaction partners (e.g., family members, friends, coworkers). This approach limits us in concluding whether individuals profit differently from family members, with whom they spend presumably more time during the initial lockdown, compared to non-household members, to whom meetings were strictly limited. Moreover, given our small sample a hypothesis-driven approach in terms of promising risk and resilience moderators were chosen, future studies with larger data sets are needed to broaden our understanding of further individual markers, such as other PRS and brain regions. Finally, since the COVID-19 infection rates were relatively low in Germany, an underestimation of the physical and mental health consequences within our sample cannot be ruled out.

Taken together, our findings highlight the protective role of social interactions on well-being both before and during the pandemic lockdown. Compared to normal times, individuals with smaller amygdala, low levels of neuroticisms, and low risk for schizophrenia showed an increased social affective benefit during social contact restrictions. In this respect, our findings suggest critical determinants of social affective gain, which might act as targets for future interventions.

## Supplementary information

Suppementary_Methods_Results

Supplementary Figure 1
