# Supplementary material for: Real-time individual benefit from social interactions before and during the lockdown: the crucial role of personality, neurobiology and genes
Source: Transl Psychiatry. 2022 Jan 21;12:28. doi: 10.1038/s41398-022-01799-z (PMC8777449; doi:10.1038/s41398-022-01799-z)
Supplement: Supplementary file 1 — Suppementary_Methods_Results [file 41398_2022_1799_MOESM1_ESM.docx]

# Supplementary Materials and Methods

## Sample representativeness

Given the longitudinal design of our study, participants are almost at equal age, representing a middle aged society at the beginning of their professional careers. In relation to the initial sample, the current sample was well balanced with regard to psychosocial risk factors (22 (31.4 %) participants without psychosocial risk, 26 (37.1%) with low psychosocial risk, and 22 (31.4%) with high psychosocial risk at birth) and gender distribution. Moreover, participants included in the present study did not systematically differ from the dropout sample with regard to educational level (T = -1.157, p = .250), income (T = 1.069, p = .287), or the experience of stressful life events (T = -.474; p = .636). When comparing our participants to publicly available reference data of the German general population, our participants show similar educational levels (current sample: highest educational degree (university) = 21.4 %; German reference sample = 28.6 %), and report a comparable financial household situation (current sample: mean monthly income = 3,880 €; German reference sample: mean monthly income = 3,580 €)^1^. In total, our current sample very well represents the German general population in this age group.

# Supplementary Results

## Supplementary Table 1.

Descriptive data

| **Descriptive data (n = 70)** | | | |
| --- | --- | --- | --- |
|  | *Mean* | *SD* | *Range* |
| Positive affect | 4.60 | 1 | 1 - 7 |
| Negative affect | 1.47 | 0.78 | 1 - 5.22 |
| COVID-19 stress burden | 7.93 | 4.84 | 2 - 18 |
| Stressful life events | 58.11 | 21.77 | 28 - 129 |
| Psychosocial Risk factors at birth | 1.87 | 1.8 | 0 - 7 |
| Neuroticism | 18.28 | 9.06 | 2 - 39 |
| SCZ-PRS | -0.0015 | 0.0001 | -0.0017 - -0.0012 |
| Left Amygdala Volume (mm³) | 1633.7 | 179.87 | 1089 - 2017 |
| Right Amygdala Volume (mm³) | 1727.1 | 204.8 | 1301 - 2176 |
| Estimated Total Intracranial Volume (mm³) | 1503564 | 128345 | 1257021 - 1932126 |
| Age (years) | 33.37 | 0.57 | 32.25 - 34.25 |

## Supplementary Table 2a.

Mixed model results for social contacts and positive affective states across both time points

|  | **Positive Affect** | | | | |
| --- | --- | --- | --- | --- | --- |
| *Predictors* | *Estimates* | *std. Beta* | *CI* | *standardized CI* | *p* |
| (Intercept) | 4.7108 | 0.0054 | 4.1600 – 5.2616 | -0.1959 – 0.2067 | <0.001 |
| Stressful life events | -0.0017 | -0.0366 | -0.0102 – 0.0069 | -0.2226 – 0.1495 | 0.700 |
| Psychosocial risk at birth | -0.0433 | -0.0776 | -0.1451 – 0.0586 | -0.2602 – 0.1050 | 0.405 |
| Gender | 0.1002 | 0.0999 | -0.2160 – 0.4163 | -0.2156 – 0.4155 | 0.535 |
| Time of day | -0.0002 | -0.0011 | -0.0081 – 0.0076 | -0.0366 – 0.0343 | 0.951 |
| Time point | -0.0738 | -0.0737 | -0.1090 – -0.0386 | -0.1088 – -0.0385 | **<0.001** |
| Momentary real-life contacts | 0.0215 | 0.1035 | 0.0087 – 0.0343 | 0.0420 – 0.1651 | **0.001** |
| Aggregated real-life contacts | 0.1343 | 0.2278 | 0.0424 – 0.2262 | 0.0719 – 0.3837 | **0.004** |
| Momentary real-life contacts * Time point | 0.0010 | 0.0047 | -0.0109 – 0.0129 | -0.0525 – 0.0620 | 0.871 |
| **Random Effects** | | | | | |
| σ^2^ | 0.4917 | | | | |
| τ_00_ _Participants_ | 0.5164 | | | | |
| τ_11_ _Time of day_ | 0.0008 | | | | |
| τ_11_ _Real-life contacts_ | 0.0018 | | | | |
| ICC | 0.50 | | | | |
| N _VPNr_ | 70 | | | | |
| Observations | 6837 | | | | |
| Marginal R^2^ / Conditional R^2^ | 0.074 / 0.540 | | | | |

## Supplementary Table 2b.

Mixed model results for social contacts and positive affective states across both time points

|  | **Positive Affect** | | | | |
| --- | --- | --- | --- | --- | --- |
| *Predictors* | *Estimates* | *std. Beta* | *CI* | *standardized CI* | *p* |
| (Intercept) | 4.7481 | -0.0343 | 4.2236 – 5.2726 | -0.2351 – 0.1665 | <0.001 |
| Stressful life events | -0.0018 | -0.0424 | -0.0099 – 0.0062 | -0.2283 – 0.1435 | 0.655 |
| Psychosocial risk at birth | -0.0360 | -0.0668 | -0.1329 – 0.0610 | -0.2467 – 0.1132 | 0.467 |
| Gender | 0.1908 | 0.1981 | -0.1096 – 0.4913 | -0.1138 – 0.5100 | 0.213 |
| Time of day | -0.0030 | -0.0138 | -0.0102 – 0.0042 | -0.0473 – 0.0196 | 0.417 |
| Time point | -0.0963 | -0.1031 | -0.1319 – -0.0607 | -0.1407 – -0.0655 | **<0.001** |
| Momentary real-life contacts | 0.0132 | 0.0693 | 0.0042 – 0.0222 | 0.0222 – 0.1165 | **0.004** |
| Quality of most important interaction | 0.0151 | 0.2466 | 0.0127 – 0.0176 | 0.2070 – 0.2862 | **<0.001** |
| Aggregated real-life contacts | 0.1186 | 0.2037 | 0.0313 – 0.2060 | 0.0537 – 0.3537 | **0.008** |
| Momentary real-life contacts * Time point | -0.0073 | -0.0383 | -0.0189 – 0.0043 | -0.0993 – 0.0228 | 0.219 |
| Quality of most important interaction * Time point | 0.0005 | 0.0078 | -0.0018 – 0.0028 | -0.0295 – 0.0452 | 0.681 |
| **Random Effects** | | | | | |
| σ^2^ | 0.4215 | | | | |
| τ_00_ _Participants_ | 0.5022 | | | | |
| τ_11_ _Time of day_ | 0.0006 | | | | |
| τ_11_ _Real-life contacts_ | 0.0005 | | | | |
| τ_11_ _Quality of most important interaction_ | 0.0001 | | | | |
| ICC | 0.52 | | | | |
| N _VPNr_ | 70 | | | | |
| Observations | 5820 | | | | |
| Marginal R^2^ / Conditional R^2^ | 0.125 / 0.576 | | | | |

## Supplementary Table 3a.

Mixed model results for social contacts and negative affective states across both time points

|  | **Negative Affect** | | | | |
| --- | --- | --- | --- | --- | --- |
| *Predictors* | *Estimates* | *std. Beta* | *CI* | *standardized CI* | *p* |
| (Intercept) | 0.7908 | -0.1166 | 0.3841 – 1.1974 | -0.3139 – 0.0807 | <0.001 |
| Stressful life events | 0.0104 | 0.2886 | 0.0041 – 0.0166 | 0.1141 – 0.4630 | **0.001** |
| Psychosocial risk at birth | 0.0343 | 0.0785 | -0.0402 – 0.1088 | -0.0921 – 0.2491 | 0.367 |
| Gender | 0.1678 | 0.2138 | -0.0635 – 0.3990 | -0.0809 – 0.5086 | 0.155 |
| Time of day | -0.0028 | -0.0161 | -0.0071 – 0.0015 | -0.0409 – 0.0087 | 0.204 |
| Time point | 0.0348 | 0.0443 | 0.0102 – 0.0593 | 0.0131 – 0.0756 | **0.005** |
| Momentary real-life contacts | -0.0035 | -0.0214 | -0.0146 – 0.0076 | -0.0898 – 0.0471 | 0.540 |
| Aggregated real-life contacts | -0.0755 | -0.1636 | -0.1424 – -0.0086 | -0.3085 – -0.0187 | **0.027** |
| Momentary real-life contacts * Time point | 0.0023 | 0.0142 | -0.0060 – 0.0106 | -0.0369 – 0.0654 | 0.585 |
| **Random Effects** | | | | | |
| σ^2^ | 0.2384 | | | | |
| τ_00_ _Participants_ | 0.3073 | | | | |
| τ_11_ _Time of day_ | 0.0002 | | | | |
| τ_11_ _Real-life contacts_ | 0.0016 | | | | |
| ICC | 0.57 | | | | |
| N _VPNr_ | 70 | | | | |
| Observations | 6837 | | | | |
| Marginal R^2^ / Conditional R^2^ | 0.136 / 0.630 | | | | |

## Supplementary Table 3a.

Mixed model results for social contacts and negative affective states across both time points

|  | **Negative Affect** | | | | |
| --- | --- | --- | --- | --- | --- |
| *Predictors* | *Estimates* | *std. Beta* | *CI* | *standardized CI* | *p* |
| (Intercept) | 0.8142 | -0.1102 | 0.4488 – 1.1795 | -0.3040 – 0.0836 | <0.001 |
| Stressful life events | 0.0095 | 0.2847 | 0.0039 – 0.0151 | 0.1173 – 0.4520 | **0.001** |
| Psychosocial risk at birth | 0.0288 | 0.0698 | -0.0378 – 0.0953 | -0.0915 – 0.2311 | 0.396 |
| Gender | 0.1733 | 0.2349 | -0.0319 – 0.3785 | -0.0433 – 0.5132 | 0.098 |
| Time of day | -0.0019 | -0.0116 | -0.0059 – 0.0021 | -0.0357 – 0.0125 | 0.345 |
| Time point | 0.0179 | 0.0254 | -0.0064 – 0.0421 | -0.0080 – 0.0588 | 0.148 |
| Momentary real-life contacts | 0.0019 | 0.0130 | -0.0043 – 0.0081 | -0.0293 – 0.0554 | 0.547 |
| Quality of most important interaction | -0.0073 | -0.1545 | -0.0092 – -0.0053 | -0.1962 – -0.1128 | **<0.001** |
| Aggregated real-life contacts | -0.0657 | -0.1474 | -0.1246 – -0.0068 | -0.2796 – -0.0153 | **0.029** |
| Momentary real-life contacts * Time point | 0.0021 | 0.0142 | -0.0057 – 0.0098 | -0.0391 – 0.0676 | 0.601 |
| Quality of most important interaction * Time point | 0.0001 | 0.0013 | -0.0015 – 0.0016 | -0.0321 – 0.0347 | 0.938 |
| **Random Effects** | | | | | |
| σ^2^ | 0.1971 | | | | |
| τ_00_ _Participants_ | 0.2848 | | | | |
| τ_11_ _Time of day_ | 0.0002 | | | | |
| τ_11_ _Real-life contacts_ | 0.0003 | | | | |
| τ_11_ _Quality of most important interaction_ | 0.0001 | | | | |
| ICC | 0.57 | | | | |
| N _VPNr_ | 70 | | | | |
| Observations | 5820 | | | | |
| Marginal R^2^ / Conditional R^2^ | 0.155 / 0.640 | | | | |

## Supplementary Table 4a.

Mixed model results for social contacts, right amygdala volume, and positive affective state across both time points

|  | **Positive Affect** | | | | |
| --- | --- | --- | --- | --- | --- |
| *Predictors* | *Estimates* | *std. Beta* | *CI* | *standardized CI* | *p* |
| (Intercept) | 4.7536 | 0.0284 | 4.1717 – 5.3355 | -0.1893 – 0.2461 | **<0.001** |
| ICV | -0.0020 | -0.0438 | -0.0107 – 0.0066 | -0.2325 – 0.1449 | 0.649 |
| Stressful life events | 0.0015 | 0.1916 | -0.0001 – 0.0031 | -0.0126 – 0.3959 | 0.066 |
| Psychosocial risk at birth | -0.0412 | -0.0739 | -0.1420 – 0.0595 | -0.2545 – 0.1067 | 0.423 |
| Gender | 0.0410 | 0.0409 | -0.3470 – 0.4289 | -0.3463 – 0.4281 | 0.836 |
| Time of day | -0.0002 | -0.0011 | -0.0081 – 0.0076 | -0.0365 – 0.0344 | 0.952 |
| Time point | -0.0746 | -0.0734 | -0.1098 – -0.0394 | -0.1085 – -0.0383 | **<0.001** |
| Right amygdala volume | -0.0005 | -0.0967 | -0.0016 – 0.0006 | -0.3184 – 0.1250 | 0.393 |
| Momentary real-life contacts | 0.0214 | 0.1033 | 0.0086 – 0.0342 | 0.0417 – 0.1649 | **0.001** |
| Aggregated real-life contacts | 0.1389 | 0.2356 | 0.0478 – 0.2299 | 0.0811 – 0.3901 | **0.003** |
| Right Amygdala * Time point | -0.0002 | -0.0468 | -0.0004 – -0.0001 | -0.0814 – -0.0122 | **0.008** |
| Momentary real-life contacts * Time point | 0.0015 | 0.0075 | -0.0104 – 0.0134 | -0.0500 – 0.0649 | 0.807 |
| Momentary real-life contacts * right amygdala volume | -0.0000 | -0.0044 | -0.0001 – 0.0001 | -0.0668 – 0.0580 | 0.890 |
| Momentary real-life contacts * right amygdala volume * Time point | -0.0000 | -0.0139 | -0.0001 – 0.0001 | -0.0790 – 0.0511 | 0.675 |
| **Random Effects** | | | | | |
| σ^2^ | 0.4915 | | | | |
| τ_00_ _Participants_ | 0.5137 | | | | |
| τ_11_ _Time of day_ | 0.0009 | | | | |
| τ_11_ _Real-life contacts_ | 0.0017 | | | | |
| ICC | 0.51 | | | | |
| N _VPNr_ | 69 | | | | |
| Observations | 6837 | | | | |
| Marginal R^2^ / Conditional R^2^ | 0.092 / 0.546 | | | | |

## Supplementary Table 4b.

Mixed model results for social contacts, right amygdala volume, and positive affective state across both time points

|  | **Positive Affect** | | | | |
| --- | --- | --- | --- | --- | --- |
| *Predictors* | *Estimates* | *std. Beta* | *CI* | *standardized CI* | *p* |
| (Intercept) | 4.6419 | -0.0302 | 4.4153 – 4.8684 | -0.2445 – 0.1842 | <0.001 |
| ICV | 0.0014 | 0.1904 | -0.0001 – 0.0029 | -0.0139 – 0.3948 | 0.068 |
| Stressful life events | -0.0023 | -0.0505 | -0.0112 – 0.0065 | -0.2416 – 0.1406 | 0.605 |
| Psychosocial risk at birth | -0.0269 | -0.0500 | -0.1239 – 0.0701 | -0.2304 – 0.1303 | 0.587 |
| Gender | 0.1824 | 0.1887 | -0.1862 – 0.5509 | -0.1927 – 0.5701 | 0.332 |
| Time of day | -0.0030 | -0.0141 | -0.0103 – 0.0043 | -0.0479 – 0.0198 | 0.416 |
| Time point | -0.0988 | -0.1022 | -0.1348 – -0.0627 | -0.1400 – -0.0643 | **<0.001** |
| Right amygdala volume | -0.0006 | -0.1417 | -0.0017 – 0.0004 | -0.3658 – 0.0824 | 0.215 |
| Momentary real-life contacts | 0.0134 | 0.0701 | 0.0041 – 0.0228 | 0.0212 – 0.1189 | **0.005** |
| Quality of most important interaction | 0.0152 | 0.2469 | 0.0128 – 0.0176 | 0.2072 – 0.2866 | **<0.001** |
| Aggregated real-life contacts | 0.1213 | 0.2080 | 0.0341 – 0.2086 | 0.0584 – 0.3575 | **0.006** |
| Right Amygdala * Time point | -0.0003 | -0.0622 | -0.0004 – -0.0001 | -0.0996 – -0.0247 | **0.001** |
| Momentary real-life contacts * Time point | -0.0073 | -0.0371 | -0.0192 – 0.0046 | -0.0995 – 0.0252 | 0.231 |
| Momentary real-life contacts * right amygdala volume | -0.000003 | -0.0004 | -0.0000 – 0.0000 | -0.0527 – 0.0518 | 0.987 |
| Quality of most important interaction * Time point | 0.0005 | 0.0074 | -0.0018 – 0.0028 | -0.0304 – 0.0452 | 0.686 |
| Quality of most important interaction * right amygdala volume | 0.000002 | 0.0118 | -0.0000 – 0.0000 | -0.0297 – 0.0533 | 0.576 |
| Momentary real-life contacts * right amygdala volume * Time point | -0.00001 | -0.0165 | -0.0001 – 0.0001 | -0.0885 – 0.0555 | 0.654 |
| Quality of most important interaction * right amygdala volume * Time point | 0.000001 | 0.0080 | -0.0000 – 0.0000 | -0.0309 – 0.0468 | 0.688 |
| **Random Effects** | | | | | |
| σ^2^ | 0.4209 | | | | |
| τ_00_ _Participants_ | 0.5006 | | | | |
| τ_11_ _Time of day_ | 0.0007 | | | | |
| τ_11_ _Real-life contacts_ | 0.0005 | | | | |
| τ_11_ _Quality of most important interaction_ | 0.0001 | | | | |
| ICC | 0.51 | | | | |
| N _VPNr_ | 69 | | | | |
| Observations | 5742 | | | | |
| Marginal R^2^ / Conditional R^2^ | 0.143 / 0.581 | | | | |

## Supplementary Table 5.

Mixed model results for social contacts, left amygdala volume, and positive affective states across both time points

|  | **Positive Affect** | | | | |
| --- | --- | --- | --- | --- | --- |
| *Predictors* | *Estimates* | *std. Beta* | *CI* | *standardized CI* | *p* |
| (Intercept) | 4.7180 | -0.0297 | 4.1863 – 5.2497 | -0.2357 – 0.1764 | <0.001 |
| ICV | 0.0016 | 0.2189 | 0.0002 – 0.0031 | 0.0234 – 0.4143 | **0.028** |
| Stressful life events | -0.0012 | -0.0272 | -0.0092 – 0.0068 | -0.2101 – 0.1558 | 0.771 |
| Psychosocial risk at birth | -0.0245 | -0.0455 | -0.1181 – 0.0691 | -0.2192 – 0.1282 | 0.608 |
| Gender | 0.1730 | 0.1796 | -0.1647 – 0.5108 | -0.1709 – 0.5302 | 0.315 |
| Time of day | -0.0030 | -0.0141 | -0.0102 – 0.0041 | -0.0474 – 0.0193 | 0.409 |
| Time point | -0.0978 | -0.1021 | -0.1334 – -0.0623 | -0.1397 – -0.0646 | **<0.001** |
| Left amygdala volume | -0.0010 | -0.1950 | -0.0021 – -0.0000 | -0.3927 – 0.0027 | **0.049** |
| Momentary real-life contacts | 0.0138 | 0.0713 | 0.0052 – 0.0223 | 0.0265 – 0.1160 | **0.002** |
| Quality of most important interaction | 0.0152 | 0.2472 | 0.0128 – 0.0176 | 0.2075 – 0.2869 | **<0.001** |
| Aggregated real-life contacts | 0.0994 | 0.1708 | 0.0141 – 0.1848 | 0.0243 – 0.3173 | **0.022** |
| Left Amygdala * Time point | -0.0003 | -0.0737 | -0.0005 – -0.0002 | -0.1110 – -0.0364 | **<0.001** |
| Momentary real-life contacts * Time point | -0.0066 | -0.0321 | -0.0182 – 0.0049 | -0.0928 – 0.0286 | 0.260 |
| Momentary real-life contacts * Left amygdala volume | 0.00003 | 0.0358 | -0.0000 – 0.0001 | -0.0120 – 0.0836 | 0.142 |
| Quality of most important interaction * Time point | 0.0004 | 0.0070 | -0.0019 – 0.0027 | -0.0304 – 0.0444 | 0.717 |
| Quality of most important interaction * Left amygdala volume | 0.000001 | 0.0150 | -0.0000 – 0.0000 | -0.0261 – 0.0562 | 0.474 |
| Momentary real-life contacts * Left amygdala volume * Time point | -0.0001 | -0.0900 | -0.0002 – -0.0000 | -0.1552 – -0.0247 | **0.007** |
| Quality of most important interaction * Left amygdala volume * Time point | -0.000001 | -0.0040 | -0.0000 – 0.0000 | -0.0430 – 0.0349 | 0.840 |
| **Random Effects** | | | | | |
| σ^2^ | 0.4201 | | | | |
| τ_00_ _Participants_ | 0.4843 | | | | |
| τ_11_ _Time of day_ | 0.0007 | | | | |
| τ_11_ _Real-life contacts_ | 0.0004 | | | | |
| τ_11_ _Quality of most important interaction_ | 0.0001 | | | | |
| ICC | 0.50 | | | | |
| N _VPNr_ | 70 | | | | |
| Observations | 5820 | | | | |
| Marginal R^2^ / Conditional R^2^ | 0.159 / 0.582 | | | | |

## Supplementary Table 6.

Mixed model results for social contacts, neuroticism, and positive affective states across both time points

|  | **Positive Affect** | | | | |
| --- | --- | --- | --- | --- | --- |
| *Predictors* | *Estimates* | *std. Beta* | *CI* | *standardized CI* | *p* |
| (Intercept) | 4.3638 | -0.0178 | 3.8327 – 4.8950 | -0.2041 – 0.1686 | <0.001 |
| Stressful life events | 0.0048 | 0.1109 | -0.0037 – 0.0134 | -0.0861 – 0.3080 | 0.270 |
| Psychosocial risk at birth | -0.0561 | -0.1067 | -0.1449 – 0.0327 | -0.2754 – 0.0621 | 0.216 |
| Gender | 0.1368 | 0.1449 | -0.1365 – 0.4101 | -0.1446 – 0.4345 | 0.327 |
| Time of day | -0.0029 | -0.0136 | -0.0102 – 0.0044 | -0.0482 – 0.0210 | 0.442 |
| Time point | -0.0865 | -0.0970 | -0.1220 – -0.0510 | -0.1353 – -0.0587 | **<0.001** |
| Neuroticism | -0.0337 | -0.3280 | -0.0507 – -0.0166 | -0.4933 – -0.1627 | **<0.001** |
| Momentary real-life contacts | 0.0161 | 0.0831 | 0.0063 – 0.0259 | 0.0328 – 0.1334 | **0.001** |
| Quality of most important interaction | 0.0151 | 0.2519 | 0.0126 – 0.0176 | 0.2106 – 0.2932 | **<0.001** |
| Aggregated real-life contacts | 0.0780 | 0.1327 | -0.0050 – 0.1610 | -0.0085 – 0.2739 | 0.066 |
| Neuroticism * Time point | 0.0051 | 0.0407 | 0.0012 – 0.0090 | 0.0029 – 0.0786 | **0.011** |
| Momentary real-life contacts * Time point | -0.0120 | -0.0606 | -0.0240 – 0.0001 | -0.1225 – 0.0013 | 0.051 |
| Momentary real-life contacts * Neuroticism | -0.0002 | -0.0117 | -0.0014 – 0.0009 | -0.0653 – 0.0419 | 0.668 |
| Quality of most important interaction * Time point | 0.0006 | 0.0097 | -0.0017 – 0.0029 | -0.0286 – 0.0479 | 0.630 |
| Quality of most important interaction * Neuroticism | 0.0001 | 0.0183 | -0.0001 – 0.0004 | -0.0217 – 0.0583 | 0.369 |
| Momentary real-life contacts * Neuroticism * Time point | -0.0022 | -0.1062 | -0.0037 – -0.0008 | -0.1732 – -0.0391 | **0.002** |
| Quality of most important interaction * Neuroticism * Time point | -0.0002 | -0.0339 | -0.0004 – 0.0000 | -0.0688 – 0.0009 | 0.056 |
| **Random Effects** | | | | | |
| σ^2^ | 0.4089 | | | | |
| τ_00_ _Participants_ | 0.3882 | | | | |
| τ_11_ _Time of day_ | 0.0007 | | | | |
| τ_11_ _Real-life contacts_ | 0.0007 | | | | |
| τ_11_ _Quality of most important interaction_ | 0.0001 | | | | |
| ICC | 0.47 | | | | |
| N _VPNr_ | 69 | | | | |
| Observations | 5742 | | | | |
| Marginal R^2^ / Conditional R^2^ | 0.183 / 0.569 | | | | |

## Supplementary Table 7.

Mixed model results for social contacts, SCZ-PRS, and positive affective states across both time points

|  | **Positive Affect** | | | | |
| --- | --- | --- | --- | --- | --- |
| *Predictors* | *Estimates* | *std. Beta* | *CI* | *standardized CI* | *p* |
| (Intercept) | 4.5638 | -0.0589 | 3.9908 – 5.1368 | -0.2697 – 0.1519 | <0.001 |
| Stressful life events | 0.0002 | 0.0046 | -0.0084 – 0.0088 | -0.1929 – 0.2022 | 0.964 |
| Psychosocial risk at birth | -0.0833 | -0.1551 | -0.1929 – 0.0262 | -0.3589 – 0.0487 | 0.136 |
| Gender | 0.2451 | 0.2533 | -0.0797 – 0.5699 | -0.0824 – 0.5890 | 0.139 |
| Time of day | -0.0020 | -0.0094 | -0.0093 – 0.0052 | -0.0428 – 0.0241 | 0.584 |
| Time point | -0.1047 | -0.1081 | -0.1408 – -0.0686 | -0.1462 – -0.0700 | **<0.001** |
| SCZ-PRS | -0.0765 | -0.0836 | -0.2389 – 0.0859 | -0.2559 – 0.0887 | 0.356 |
| Momentary real-life contacts | 0.0135 | 0.0719 | 0.0043 – 0.0226 | 0.0234 – 0.1204 | **0.004** |
| Quality of most important interaction | 0.0151 | 0.2481 | 0.0127 – 0.0175 | 0.2080 – 0.2883 | **<0.001** |
| Aggregated real-life contacts | 0.1022 | 0.1753 | 0.0007 – 0.2036 | 0.0012 – 0.3494 | **0.048** |
| SCZ-PRS * Time point | -0.0732 | -0.0817 | -0.1075 – -0.0389 | -0.1187 – -0.0447 | **<0.001** |
| Momentary real-life contacts * Time point | -0.0027 | -0.0135 | -0.0149 – 0.0095 | -0.0782 – 0.0511 | 0.663 |
| Momentary real-life contacts * SCZ-PRS | -0.0053 | -0.0289 | -0.0137 – 0.0031 | -0.0744 – 0.0167 | 0.214 |
| Quality of most important interaction * Time point | 0.0004 | 0.0068 | -0.0019 – 0.0027 | -0.0309 – 0.0446 | 0.741 |
| Quality of most important interaction * SCZ-PRS | 0.0013 | 0.0223 | -0.0011 – 0.0037 | -0.0178 – 0.0623 | 0.275 |
| Momentary real-life contacts * SCZ-PRS * Time point | -0.0088 | -0.0481 | -0.0188 – 0.0012 | -0.1025 – 0.0063 | 0.083 |
| Quality of most important interaction * SCZ-PRS * Time point | -0.0018 | -0.0309 | -0.0039 – 0.0003 | -0.0661 – 0.0043 | 0.085 |
| **Random Effects** | | | | | |
| σ^2^ | 0.4179 | | | | |
| τ_00_ _Participants_ | 0.4835 | | | | |
| τ_11_ _Time of day_ | 0.0006 | | | | |
| τ_11_ _Real-life contacts_ | 0.0005 | | | | |
| τ_11_ _Quality of most important interaction_ | 0.0001 | | | | |
| ICC | 0.51 | | | | |
| N _VPNr_ | 68 | | | | |
| Observations | 5631 | | | | |
| Marginal R^2^ / Conditional R^2^ | 0.188 / 0.604 | | | | |

# Sensitivity analyses

## Supplementary Table 8.

Mixed model results for social contacts, left amygdala volume, and positive affective states across both time points after excluding one participant, who underwent EMA procedures in February 2020.

|  | **Positive Affect** | | | | |
| --- | --- | --- | --- | --- | --- |
| *Predictors* | *Estimates* | *std. Beta* | *CI* | *standardized CI* | *p* |
| (Intercept) | 4.7031 | 0.0196 | 4.1387 – 5.2674 | -0.1920 – 0.2313 | <0.001 |
| ICV | -0.0015 | -0.0332 | -0.0100 – 0.0070 | -0.2199 – 0.1535 | 0.727 |
| Stressful life events | 0.0015 | 0.1993 | -0.0000 – 0.0031 | -0.0016 – 0.4002 | 0.052 |
| Psychosocial risk at birth | -0.0274 | -0.0492 | -0.1272 – 0.0725 | -0.2287 – 0.1303 | 0.591 |
| Gender | 0.0617 | 0.0618 | -0.3010 – 0.4243 | -0.3014 – 0.4250 | 0.739 |
| Time of day | -0.0005 | -0.0025 | -0.0085 – 0.0074 | -0.0386 – 0.0336 | 0.892 |
| Time point | -0.0692 | -0.0682 | -0.1047 – -0.0338 | -0.1037 – -0.0326 | **<0.001** |
| Left amygdala volume | -0.0009 | -0.1603 | -0.0020 – 0.0002 | -0.3593 – 0.0387 | 0.114 |
| Momentary real-life contacts | 0.0215 | 0.1038 | 0.0088 – 0.0342 | 0.0420 – 0.1656 | **0.001** |
| Aggregated real-life contacts | 0.1253 | 0.2147 | 0.0345 – 0.2161 | 0.0591 – 0.3703 | **0.007** |
| Left Amygdala * Time point | -0.0003 | -0.0524 | -0.0005 – -0.0001 | -0.0875 – -0.0172 | **0.004** |
| Momentary real-life contacts * Time point | 0.0010 | 0.0068 | -0.0109 – 0.0130 | -0.0514 – 0.0649 | 0.868 |
| Momentary real-life contacts * Left amygdala volume | 0.0000 | 0.0415 | -0.0000 – 0.0001 | -0.0217 – 0.1046 | 0.198 |
| Momentary real-life contacts * Left amygdala volume * Time point | -0.0001 | -0.0802 | -0.0002 – -0.0000 | -0.1399 – -0.0206 | **0.008** |
| **Random Effects** | | | | | |
| σ^2^ | 0.4919 | | | | |
| τ_00_ _Participants_ | 0.4973 | | | | |
| τ_11_ _Time of day_ | 0.0008 | | | | |
| τ_11_ _Real-life contacts_ | 0.0002 | | | | |
| ICC | 0.49 | | | | |
| N _VPNr_ | 69 | | | | |
| Observations | 6745 | | | | |
| Marginal R^2^ / Conditional R^2^ | 0.101 / 0.541 | | | | |

## Supplementary Table 9.

Mixed model results for social contacts, neuroticism, and positive affective states across both time points after excluding one participant, who underwent EMA procedures in February 2020.

|  | **Positive Affect** | | | | |
| --- | --- | --- | --- | --- | --- |
| *Predictors* | *Estimates* | *std. Beta* | *CI* | *standardized CI* | *p* |
| (Intercept) | 4.2526 | 0.0083 | 3.7075 – 4.7976 | -0.1792 – 0.1957 | <0.001 |
| Stressful life events | 0.0060 | 0.1333 | -0.0028 – 0.0149 | -0.0623 – 0.3290 | 0.182 |
| Psychosocial risk at birth | -0.0611 | -0.1128 | -0.1530 – 0.0308 | -0.2825 – 0.0569 | 0.193 |
| Gender | 0.0718 | 0.0735 | -0.2092 – 0.3527 | -0.2142 – 0.3612 | 0.617 |
| Time of day | -0.0003 | -0.0012 | -0.0083 – 0.0078 | -0.0386 – 0.0362 | 0.951 |
| Time point | -0.0675 | -0.0674 | -0.1027 – -0.0323 | -0.1034 – -0.0315 | **<0.001** |
| Neuroticism | -0.0385 | -0.3668 | -0.0566 – -0.0203 | -0.5398 – -0.1937 | **<0.001** |
| Momentary real-life contacts | 0.0242 | 0.1167 | 0.0107 – 0.0378 | 0.0520 – 0.1814 | **<0.001** |
| Aggregated real-life contacts | 0.0867 | 0.1469 | 0.0019 – 0.1715 | 0.0032 – 0.2907 | **0.045** |
| Neuroticism * Time point | 0.0050 | 0.0473 | 0.0012 – 0.0087 | 0.0114 – 0.0833 | **0.010** |
| Momentary real-life contacts * Time point | -0.0047 | -0.0260 | -0.0170 – 0.0076 | -0.0844 – 0.0325 | 0.453 |
| Momentary real-life contacts * Neuroticism | 0.0007 | 0.0319 | -0.0008 – 0.0022 | -0.0345 – 0.0983 | 0.347 |
| Momentary real-life contacts * Neuroticism * Time point | -0.0023 | -0.1001 | -0.0037 – -0.0008 | -0.1628 – -0.0375 | **0.002** |
| **Random Effects** | | | | | |
| σ^2^ | 0.4754 | | | | |
| τ_00_ _Participants_ | 0.3975 | | | | |
| τ_11_ _Time of day_ | 0.0008 | | | | |
| τ_11_ _Real-life contacts_ | 0.0020 | | | | |
| ICC | 0.46 | | | | |
| N _VPNr_ | 68 | | | | |
| Observations | 6644 | | | | |
| Marginal R^2^ / Conditional R^2^ | 0.142 / 0.539 | | | | |

## Supplementary Table 10.

Mixed model results for social contacts, SCZ-PRS, and positive affective states across both time points after excluding one participant, who underwent EMA procedures in February 2020.

|  | **Positive Affect** | | | | |
| --- | --- | --- | --- | --- | --- |
| *Predictors* | *Estimates* | *std. Beta* | *CI* | *standardized CI* | *p* |
| (Intercept) | 4.4540 | -0.0322 | 3.8634 – 5.0445 | -0.2452 – 0.1809 | <0.001 |
| Stressful life events | 0.0011 | 0.0233 | -0.0078 – 0.0099 | -0.1723 – 0.2188 | 0.816 |
| Psychosocial risk at birth | -0.0840 | -0.1514 | -0.1980 – 0.0299 | -0.3567 – 0.0539 | 0.148 |
| Gender | 0.1835 | 0.1831 | -0.1504 – 0.5174 | -0.1501 – 0.5163 | 0.281 |
| Time of day | 0.0003 | 0.0013 | -0.0077 – 0.0083 | -0.0348 – 0.0374 | 0.945 |
| Time point | -0.0761 | -0.0755 | -0.1121 – -0.0402 | -0.1114 – -0.0396 | **<0.001** |
| SCZ-PRS | -0.0875 | -0.0877 | -0.2597 – 0.0848 | -0.2604 – 0.0850 | 0.320 |
| Momentary real-life contacts | 0.0220 | 0.1078 | 0.0088 – 0.0351 | 0.0433 – 0.1724 | **0.001** |
| Aggregated real-life contacts | 0.1009 | 0.1728 | -0.0036 – 0.2053 | -0.0061 – 0.3517 | 0.058 |
| SCZ-PRS * Time point | -0.0792 | -0.0794 | -0.1146 – -0.0439 | -0.1148 – -0.0440 | **<0.001** |
| Momentary real-life contacts * Time point | 0.0055 | 0.0273 | -0.0068 – 0.0178 | -0.0330 – 0.0876 | 0.380 |
| Momentary real-life contacts * SCZ-PRS | -0.0073 | -0.0359 | -0.0201 – 0.0055 | -0.0989 – 0.0270 | 0.263 |
| Momentary real-life contacts * SCZ-PRS * Time point | -0.0108 | -0.0533 | -0.0210 – -0.0006 | -0.1034 – -0.0032 | **0.037** |
| **Random Effects** | | | | | |
| σ^2^ | 0.4909 | | | | |
| τ_00_ _Participants_ | 0.5203 | | | | |
| τ_11_ _Time of day_ | 0.0009 | | | | |
| τ_11_ _Real-life contacts_ | 0.0018 | | | | |
| ICC | 0.50 | | | | |
| N _VPNr_ | 67 | | | | |
| Observations | 6554 | | | | |
| Marginal R^2^ / Conditional R^2^ | 0.134 / 0.571 | | | | |

## Supplementary Table 11.

Mixed model results for social contacts and positive affective states across both time points using an updated PRS for schizophrenia^2^.

|  | **Positive Affect** | | | | |
| --- | --- | --- | --- | --- | --- |
| *Predictors* | *Estimates* | *std. Beta* | *CI* | *standardized CI* | *p* |
| (Intercept) | 4.5358 | -0.0184 | 3.9304 – 5.1411 | -0.2290 – 0.1922 | <0.001 |
| Stressful life events | 0.0001 | 0.0026 | -0.0089 – 0.0092 | -0.1955 – 0.2007 | 0.979 |
| Psychosocial risk at birth | -0.0777 | -0.1395 | -0.1914 – 0.0360 | -0.3437 – 0.0647 | 0.181 |
| Gender | 0.1593 | 0.1584 | -0.1756 – 0.4942 | -0.1745 – 0.4913 | 0.351 |
| Time of day | 0.0005 | 0.0025 | -0.0073 – 0.0084 | -0.0331 – 0.0380 | 0.891 |
| Time point | -0.0815 | -0.0810 | -0.1172 – -0.0458 | -0.1165 – -0.0455 | **<0.001** |
| SCZ-PRS | -0.1155 | -0.1148 | -0.2940 – 0.0631 | -0.2923 – 0.0627 | 0.205 |
| Momentary real-life contacts | 0.0220 | 0.1068 | 0.0085 – 0.0355 | 0.0413 – 0.1723 | **0.001** |
| Aggregated real-life contacts | 0.1065 | 0.1805 | 0.0006 – 0.2125 | 0.0010 – 0.3601 | **0.049** |
| SCZ-PRS * Time point | -0.0127 | -0.0126 | -0.0475 – 0.0222 | -0.0473 – 0.0221 | 0.476 |
| Momentary real-life contacts * Time point | 0.0027 | 0.0132 | -0.0093 – 0.0148 | -0.0454 – 0.0718 | 0.657 |
| Momentary real-life contacts * SCZ-PRS | -0.0013 | -0.0062 | -0.0157 – 0.0131 | -0.0761 – 0.0637 | 0.863 |
| Momentary real-life contacts * SCZ-PRS * Time point | -0.0082 | -0.0398 | -0.0220 – 0.0056 | -0.1066 – 0.0271 | 0.244 |
| **Random Effects** | | | | | |
| σ^2^ | 0.4914 | | | | |
| τ_00_ _Participants_ | 0.5135 | | | | |
| τ_11_ _Time of day_ | 0.0008 | | | | |
| τ_11_ _Real-life contacts_ | 0.0020 | | | | |
| ICC | 0.51 | | | | |
| N _VPNr_ | 68 | | | | |
| Observations | 6646 | | | | |
| Marginal R^2^ / Conditional R^2^ | 0.142 / 0.577 | | | | |

# Supplementary Figure legends

## Supplementary Figure 1: Assessment and COVID-19 overview.

Schematic timeline of the baseline and COVID-19 ecological momentary assessment. Baseline assessment started in the beginning of 2019 and was interrupted by the COVID-19 pandemic in March 2020. The COVID-19 assessment started around one month after the initial lockdown in Germany was governmentally launched.

# References

1. Statistisches Bundesamt. Nettoeinkommen privater Haushalte nach Haushaltstyp 2020. (2020).

2. The Schizophrenia Working Group of the Psychiatric Genomics, C., Ripke, S., Walters, J.T. & O’Donovan, M.C. Mapping genomic loci prioritises genes and implicates synaptic biology in schizophrenia. *medRxiv*, 2020.2009.2012.20192922 (2020).
