# Supplementary figures and images for: Real-time individual benefit from social interactions before and during the lockdown: the crucial role of personality, neurobiology and genes
Source: Transl Psychiatry. 2022 Jan 21;12:28. doi: 10.1038/s41398-022-01799-z (PMC8777449; doi:10.1038/s41398-022-01799-z)

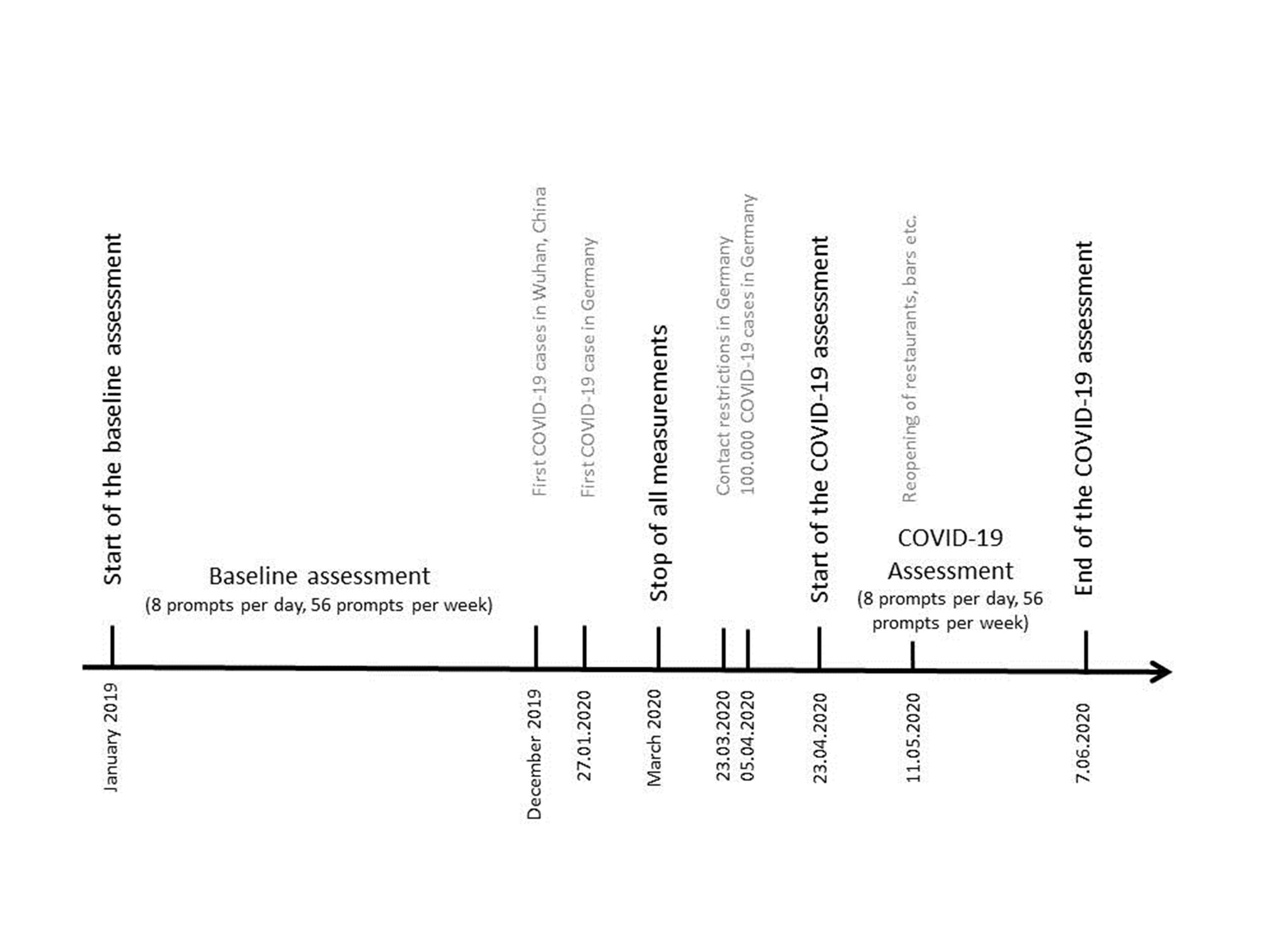

Supplement: Supplementary file 2 — Supplementary Figure 1 [file 41398_2022_1799_MOESM2_ESM.tif]
